# Medical treatment of miscarriage using misoprostol—a retrospective study

**DOI:** 10.1007/s00404-024-07628-6

**Published:** 2024-08-13

**Authors:** Laura Meister, Ines Künnemann, Franziska Fettke, Anke Lux, Atanas Ignatov

**Affiliations:** 1 Otto-von-Guericke-Universitat Magdeburg, Leibnizstrasse 43, 39104 Magdeburg, Germany; 2 Magdeburg, Deutschland; 3 Halle, Deutschland; 4 Braunschweig, Deutschland

**Keywords:** Early miscarriage, Missed abortion, Misoprostol, Double endometrial stripe thickness

## Abstract

**Purpose:**

The treatment of early miscarriage with medication is effective and low in side effects. Nevertheless, no uniform dosage regimen has yet been established, nor has it been possible to determine whether previous pregnancies and births with their respective modes of delivery play a role in the effectiveness of Misoprostol. This study aimed to find predictive parameters for successful treatment with Misoprostol in early miscarriage.

**Methods:**

In a retrospective study at the Otto von Guericke University Women’s Hospital, records of patients with early miscarriage and medical treatment using Misoprostol from 2018 to 2021 were reviewed for this purpose. The need for a curettage subsequent to treatment was scored as a parameter of failure. The data were analyzed using Statistical Package for the Social Science Version 28.0. The significance level was set to 0.050.

**Results:**

We found that successful therapy with misoprostol was seen in 86% (*n* = 114). 14% (*n* = 20) of the patients had curettage after taking Misoprostol as advised. Out of 134 women, 16% (*n* = 21) reported mild side effects, with nausea as the leading one (9.2% (*n* = 12)). Significance was found comparing the measurement of double endometrial stripe thickness after the second cycle of Misoprostol in women with and without curettage after medical treatment (exact value two-sided 0.035 at *p* < 0.05). A cutoff value at 8.8 mm was calculated using ROC Analysis.

**Conclusions:**

Our results indicate that the treatment of early miscarriage in the first trimester with Misoprostol is effective and has few side effects. The measurement of the endometrial stripe thickness after the second cycle of Misoprostol via transvaginal ultrasound could present a predictive marker during therapy.

## What does this study add to the clinical work

**Table:** 

This study adds that there is evidence of differential response to medical treatment with Misoprostol depending on whether the patient has a history of vaginal birth(s) or delivery by cesarean section. A corresponding history before administration could be of critical importance. In addition, the double endometrial stripe thickness measured after each application of Misoprostol can be seen as a predictor to success of therapy.	

## Introduction

At least 12% of all pregnancies end in miscarriage (up to 12 + 0 GA/ < 84 days) [1]. Missed abortion is defined as non-developing pregnancy with embryo, embryonic tissue, fetus or empty gestation sac remaining in utero, closed cervical os, without cardiac activity seen in ultrasound and with or without present bleeding [2].

Treatment options include the surgical management using suction curettage, as well as medical treatment with Misoprostol or expectant management. For early miscarriage, the surgical management is currently the most common procedure, although only about 10% of women have no other medical choice due to excessive bleeding, anemia, or infection [3, 4]. However, increasing cesarean section rates [5] and in vitro fertility treatment [6] represent a high number of additional surgical procedures. Any further invasive procedure can be potentially harmful to uterine structures especially the endometrium and may cause intrauterine adhesions [7] and even an Asherman syndrome [8]. Thus, increasing numbers of intrauterine surgical procedure can be associated with implantation failure or abnormal invasive placentation in further pregnancies[7].

The off-label-use drug Misoprostol is a good alternative to a suction curettage. It is inexpensive, easy to store, and several studies proved it to be safe with few side effects [9, 10] such as nausea, dizziness and diarrhea. Nevertheless, there is no standardized administration regime, including dosing, despite various guideline recommendations differing between 400 and 800 µg per dose.

So far, it has not been fully determined which patient collective should be advised to take the medical route and which should be discouraged. It is unclear if the number of previous pregnancies and their respective modes of delivery could hereby play a role.

In this study, we ask how many women succeed in treatment, which side effects occur, whether the past medical history of pregnancies and delivery modes and other parameters do have an impact to the outcome and the relation to post drug curettages. To answer this question, we retrospectively reviewed our in-house results from 2018 to 2021.

## Materials and methods

### Materials

#### Study design

To determine a correlation between anamnestic parameters and the success of therapy, we retrospectively evaluated cases at Magdeburg University Hospital (Saxony-Anhalt a federal state of Germany).

Pre-specified parameters included age, gestational age, weight in kg, height in cm, BMI, previous pregnancies, previous delivery mode, previous miscarriage or planned abortion, other uterine surgeries, general medical illness, bleeding before Misoprostol use, bleeding after Misoprostol use, double endometrial thickness per ultrasound, Hb (hemoglobin) values, side effects, pre-existing conditions, pre-existing thyroid disease, medication use, smoking, alcohol abuse, occupation, and blood group.

The study was approved by the Research and Ethical Committee of the Otto-von-Guericke University Magdeburg (Germany).

All patients obtained written informed consent before treatment. An additional individual consent for this analysis was not necessary.

#### Study participants

Collecting data took place from 2018 to end of 2021 in which 156 patient records were included. Inclusion criteria defined by all women with an ultrasound-diagnosed preterm abortion (up to 12 + 0 GA/ < 84 days) who opted for medical therapy using Misoprostol and had no contraindications to the drug. Exclusions included induction of labor and planned abortion using misoprostol, misoprostol for priming for surgical procedures, ectopic pregnancy, trophoblastic disease, haemodynamic instability, excessive vaginal bleeding, coagulopathy, septic abortion, and contraindications or hypersensitivity to the drug.

#### Procedures

**Fig. 1:**
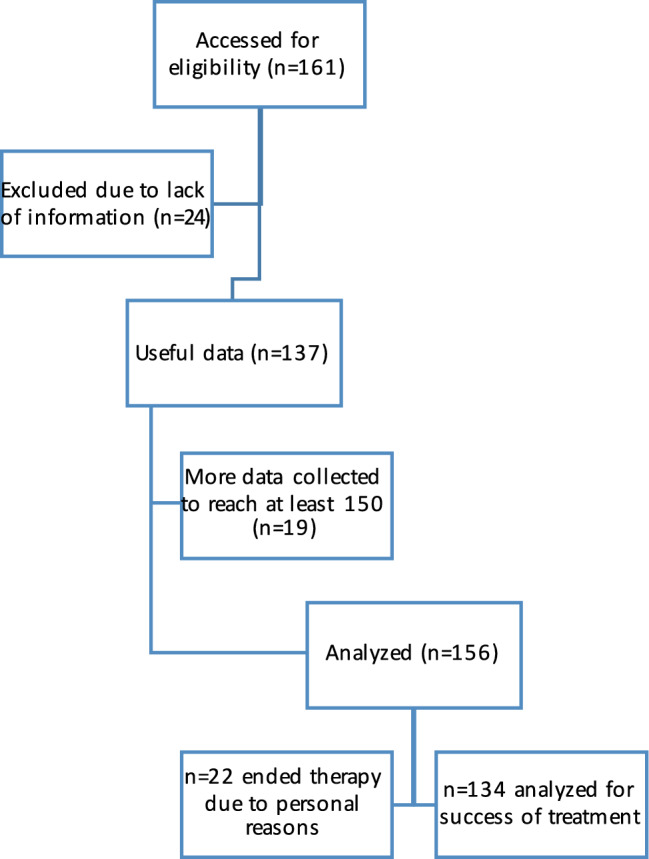
Flowchart of study participants

All eligible patients were treated according to the following protocol: The first cycle consisting of two pills Misoprostol 200 µg (400 µg) orally a.m. and p.m. given for 3 days followed by weekly ultrasound and follow-up care for the span of three cycles, representing a 3 week period in total. Depending on the outcome, the number of cycles taken are individual and differ between one and four cycles. After each cycle and following examination and recommendation by the clinician, the patients can decide if they want to proceed or not, meaning that non-medical justified reasons can also lead to the ending of medical treatment. The primary outcome measure of this study was successful treatment. Meaning abortus completus, defined as the total expulsion of any residual tissue measured via weekly ultrasound and standardized gynecological examination. Prolonged bleeding and retained products of conception need clinical action, so ultrasound represents the first-line imagining modality. Grayscale ultrasound can detect thickened endometrial lining, as shown in per example in Fig. 1.

The secondary outcome measure was the assessment of side effects reported by the women. Thereby we focused on the main side effects to Misoprostol which are nausea and vomiting, vertigo, diarrhea, headaches, shivering and fever. Information regarding side effects were documented during the follow up exams in their medical records. In addition, we focused on finding predictive parameters to success of therapy, including previous birthing modes of delivery and physical parameters. Finally, the potential predictors of complete abortion were obtained from the patient at every follow up examination or contacting patients by telephone.

#### Methods/statistics

The present retrospective analysis was used to explore the potential predictors for complete abortion in women treated with Misoprostol. All targeted parameters were individually gathered from patient medical records from the outpatient clinic and then recorded via Windows Excel.

The statistics were calculated using SPSS Version 28.0. Statistical analysis was performed with one-sided and a two-sided tests using Wilcoxon rank-sum test, Chi-Square test and Fisher’s exact test to compare the classified variables at a 5% significance level.

## Results

**Fig. 2:**
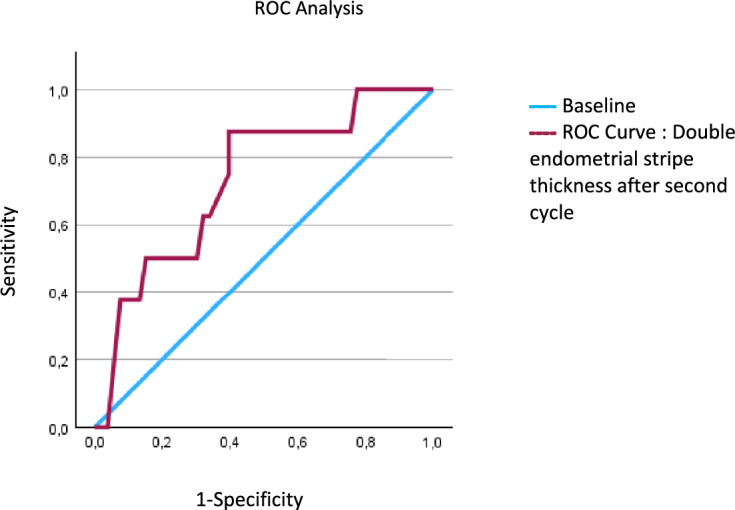
ROC-analysis double endometrial stripe thickness

Out of 156 women, 134 met the eligibility criteria for data analysis (Fig. 2).

The average age of the patients was 32 years, mean gestational age was 9 + 4 weeks (determined from the last menstrual period) and average weight was 74.9 kg (Table 1). In total 46.2% (*n* = 60) of the patients were nulliparous and 60% (*n* = 12) out of all women who underwent curettage after Misoprostol therapy were also nulliparous. 53.8% (*n* = 70) previously delivered, 46.5% (*n* = 60) vaginally and 9.9% (*n* = 13) via cesarean. 33% (*n* = 44) claimed to have had miscarriage in their past medical history. 66.7% (*n* = 88) of the women showed signs of vaginal bleeding before commencing therapy. 31.8%(*n* = 42) patients showed secondary illnesses. The mean hemoglobin value was 7.8 mmol/l. The most common blood group was A with 39.7% (*n* = 52). 17.6% (*n* = 22) were active smokers and no one showed alcohol abusive behavior. 82.3% (*n* = 102) were employed. In average the patients received 2.44 cycles of Misoprostol with one cycle being the minimum and four cycles being the maximum (Table 1).

**Table 1:** Investigated predicitive parameters (reduced patient collective)

Parameters, *n*	Abrasio *p* value Yes (*n* = 20)	No (*n* = 114)	*p* value
Age (20/114)	32.75 ± 4.34 (32.5)	32.17 ± 6.59 (32.5)	0.817
Gestational age (20/108)	10.11 ± 1.72 (9.9)	9.58 ± 1.72 (9.6)	0.284
Weight (kg) (18/34)	70.67 ± 19.85 (67.5)	77.18 ± 20.89 (70.5)	0.211
Height (cm) (18/35)	163.83 ± 5.67 (163.0)	167.77 ± 5.92 (168.0)	0.027
BMI (18/34)	26.45 ± 8.05 (22.8)	27.50 ± 7.39 (24.4)	0.290
Previous pregnancies	19	108	0.489
Previous delivery mode			
Vaginal birth	7	53	0.261
Ceasarean	1	12	0.690
Previous miscarriage	9	35	0.240
Other uterine surgeries	3	24	0.565
General medical illness	9	33	0.169
Bleeding before misoprostol	11	77	0.230
Double endometrial thickness (per ultrasound)			
Before misoprostol (1/34)	23.00	14.3 ± 580 (13)	–
After 1 cycle (7/69)	14.36 ± 8.14 (12.0)	10.84 ± 4.59 (11.0)	0.384
After 2 cycles (8/53)	11.74 ± 4.14 (11.5)	8.52 ± 4.95 (7.9)	0.035
After 3 cycles (9/33)	13.47 ± 7.78 (13.7)	8.50 ± 3.48 (8.0)	0.063
Hemoglobin value mmol/l	7.54 ± 0.92 (8.0)	7.93 ± 0.96 (8.0)	0.171
Side effects			
Nausea	2	10	1.000
Vomiting	1	2	0.394
Headache	1	2	0.394
Vertigo	1	7	1.000
Shivering	0	2	1.000
Diarrhea	2	6	0.352
Fever	1	1	0.283
Pre-existing thyroid disease	4	20	0.475
Blood pressure medication	1	3	0.483
Smoking	7	15	0.048
Alcohol abuse	0	0	–
			0.023
Employed	20	82	
Unemployed	0	22	
Blood group			0.260
A	8	42	
B	5	47	
AB	1	6	
O	6	16	

Values represent mean and SD. The *p* value represents the comparison between abrasio and non-abrasio

Our primary outcome was defined as successful treatment meaning complete abortion after medical therapy. This happened in 86% (*n* = 114) out of 134 patients. 14% (*n* = 20) underwent curettage due to insufficient expulsion of uterine material.

**Fig. 3:**
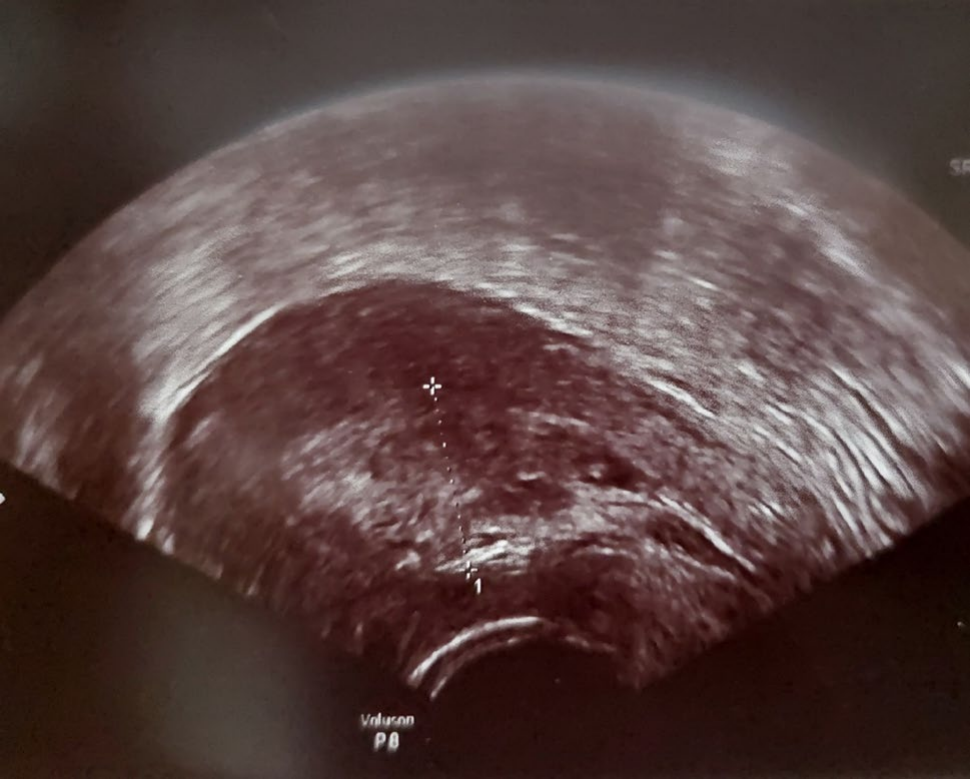
Endometrial stripe thickness per ultrasound

Our secondary outcome included the assessment of side effects to treatment. In total, out of 134 patients only 16% reported side effects to the drug, the most frequent one being nausea with 9.2%(12) following vertigo (6.1%; 8), diarrhea (6.1%; 8), vomiting (2.3%; 3), headaches (2.3%; 3), shivering (1.5%; 2) and fever (1.5%; 2). Significance was found measuring the double endometrial stripe thickness after the second cycle of Misoprostol in women who later underwent curettage compared to women without curettage after medical treatment (exact significance two-sided 0.035 at alpha = 0.05) (Fig. 3). Using ROC Analysis, we calculated the optimal cutoff value of 8,8 mm in double endometrial stripe thickness to predict a positive curettage result after the second misoprostol cycle, providing a sensitivity of 87.5% and a specificity of 60.4%.

## Discussion

In this study, we found that 86% of the women succeeded in the treatment of early miscarriage with Misoprostol. Only 16% of the patients showed side effects with nausea being the most frequent one (followed by vertigo, diarrhea, vomiting, headaches and fever) making Misoprostol safe, efficient and low in side effects for early miscarriage.

This aligns with findings from two Cochrane analyses [11, 12] which also state that it shows high patient satisfaction besides being a safe and effective method. Similar results are seen in a systematic review from 2020 [13]. Although the study focuses on medical induced abortions, which was an exclusion criterion in our study, and the fact that the studies mentioned above compared the effects of misoprostol and mifepristone taken together, it nevertheless gives an outlook to safety in various medical indications. Another review from 2021 [14] reaches similar conclusions and success was seen in 88 to 93%. However, in this study, Mifepristone 200 mg was administered 24–48 h before Misoprostol and Misoprostol taken sublingually or vaginally instead of orally, hence a comparison is only partially possible.

The various administration routes of Misoprostol present another interesting outlook. In our study, we only administered the drug orally due to psychological concerns of taking the vaginal route while having an abortion, the pill not dissolving properly due to bleeding or not dissolving sublingually because of the big size and its overall manageability. In different guidelines such as the WHO, AWMF, FIGO and NICE, Misoprostol for early abortion is given differently. The WHO [15] suggests oral or sublingual route for incomplete abortion, AWMF [16] suggests vaginal, sublingual or buccal, FIGO [17] suggests vaginal or sublingual for missed abortion and eventually oral for incomplete abortion and NICE [18] suggests vaginal or oral route if wished by the patient. A recent randomized controlled trial from 2019 [19] comparing the sublingual versus vaginal route of Misoprostol for first trimester missed abortion came to the conclusion that the sublingual route was significantly more successful in complete abortion than the vaginal route with significant shorter length of induction–expulsion time than the vaginal group (12.3 + − 3.1 h vs. 16.4 + − 4.2 h; *p* < 0,001). It did state though, that side effects occurred significantly more frequent in the sublingual arm than in the vaginal.

In an older RCT [20], the oral route was not more effective than the vaginal route but the side effects were significantly more frequent in the oral group, thus emphasizing the results from 2019.

In terms of safety and manageability of taking Misoprostol in an outpatient setting a recent national cohort study from BJOG [21] compared the outcome of early medical abortion without ultrasound via telemedicine by administering Mifepristone and Misoprostol sublingual, vaginal or buccal. They found out that it has no disadvantage to the patients taking Misoprostol in an outpatient setting and it is found to be effective and safe. However, this study only focused on induced abortions and had the possibility of providing the patients with a 24 h hotline in case of unusual events, concerns and emergencies which can be problematic to some clinics in terms of finances or staff shortages, but it does state an interesting foresight to the future of telemedicine.

The significance we found in double endometrial stripe thickness after the second cycle of Misoprostol in women who later underwent curettage compared to women without curettage (*p* = 0.035) could not only delivery a possible prognostic marker but is also in align with findings in the literature. In a retrospective study [25], they found that every additional millimeter of endometrial stripe thickness led to an additional 8% chance of undergoing further surgical procedure. It should be mentioned though, that the study not only included miscarriage but also induced abortions, vaginal deliveries and caesarean sections with material retention, hence a direct comparison is only partially possible.

Although the results in our study did not reach statistical significance, the two-sided Chi-square test showed a tendency towards more frequent secondary curettages after being treated with Misoprostol in women with increasing number of cesareans in past medical history (exact value two-sided *p* = 0.083). Further tendencies could also be seen in women with cesarean deliveries in the past medical history and the number of Misoprostol cycles undertaken. Not statistical significant, still there seems to be more frequent secondary curettages with only one cycle of Misoprostol administered (exact value two-sided 0.057 at *p* < 0.05) meaning premature termination of therapy as wished by the patients. It is needed to mention, that our results could only be found in a population of only 6 patients and an analysis to subgroups were not possible. It represents an interesting subject, which should be analyzed in larger study groups to offer a guideline to clinicians. Nonetheless, our findings are in line with the conclusions from an observational 10 year study [22] which found out that there is a significant decrease in successful treatment with misoprostol depending on previous cesarean sections (*p* = 0.002) and parity (*p* = 0.048) of the women. The history of one or more cesarean sections led to significantly more failure in medical treatment (*p* = 0.001). This is confirmed by a recent RCT from 2021 [23] stating that history of cesarean sections lead to more failure in treatment. Correspondingly a recent study from 2022 [24] examined predictive parameters to failure in treatment with Misoprostol in women with one or more cesarean sections in the past. It showed that the width and the blood flow signal of the residual seen via ultrasound were both independent predictors for complete abortion (*p* =  < 0.05). The rate of complete miscarriage was higher in women without blood flow shown in the ultrasound and residue of less than 1 cm.

All of the above findings should be considered when treating patients in the future.

The strengths of our study are: (1) minimal loss of follow-up regarding complete abortion or secondary curettage and (2) systematically performed follow-up examination in house in a standardized manner.

The retrospective character and thus leading to recall bias of the patients, especially regarding side effects (insufficient information given by the patients) represent limitation of our study. Furthermore, when admitting the patient, the individual and personal preferences to surgical or non-surgical procedure both of the clinician and the patient could present another bias. Another possible bias could be seen in clinicians treating patients who decided to end therapy due to personal reasons, as seen in our study. Because of the hospital setting for therapy and hence a more neutral relationship to the patients compared to the doctor’s office of their personal gynecologist, the bias could be limited. However, an individual approach is needed to find out which patients provide enough personal and physical resources to stick to therapy and not end it with an unsatisfying outcome. Because of recall bias, it is possible that individual findings to side effects are left unmentioned in follow-up examinations. In every follow-up visitation, we collected at least one verbal answer from every patient and the results are similar to aforementioned studies. Our results describe no correlation between parity or previous vaginal deliveries and the success of Misoprostol. Still, our study strengthens former results from other studies regarding the effectivity and safety and gives additional assumption that previous pregnancies and deliveries could still have an impact to the outcome.

In conclusion, our study provides evidence that Misoprostol is safe and has few side effects. An increased value of double endometrial stripe thickness after the second cycle of Misoprostol therapy is associated with more frequent curettage. When administering patients to follow-up exams, an intensified view to the measured endometrial stripe thickness via ultrasound could offer early possibilities to alter clinical action and thereby provide better individual therapy. Our study did not show any relation between previous pregnancies, their delivery mode and the success of therapy using Misoprostol, so future research is needed to examine these parameters in larger clinical studies.

## Data Availability

Data are available on reasonable request.
